# The sentinel node invasion level (SNIL) as a prognostic parameter in melanoma

**DOI:** 10.1038/s41379-021-00835-5

**Published:** 2021-06-15

**Authors:** Lutz Kretschmer, Christina Mitteldorf, Simin Hellriegel, Andreas Leha, Alexander Fichtner, Philipp Ströbel, Michael P. Schön, Felix Bremmer

**Affiliations:** 1 Department of Dermatology, Venereology and Allergology, University Medical Center, Göttingen, Germany; 2 Department of Medical Statistics, University Medical Center, Göttingen, Germany; 3 Institute of Pathology, University Medical Center, Göttingen, Germany

**Keywords:** Risk factors, Melanoma

## Abstract

Sentinel lymph node (SN) tumor burden is becoming increasingly important and is likely to be included in future N classifications in melanoma. Our aim was to investigate the prognostic significance of melanoma infiltration of various anatomically defined lymph node substructures. This retrospective cohort study included 1250 consecutive patients with SN biopsy. The pathology protocol required description of metastatic infiltration of each of the following lymph node substructures: intracapsular lymph vessels, subcapsular and transverse sinuses, cortex, paracortex, medulla, and capsule. Within the SN with the highest tumor burden, the SN invasion level (SNIL) was defined as follows: SNIL 1 = melanoma cells confined to intracapsular lymph vessels, subcapsular or transverse sinuses; SNIL 2 = melanoma infiltrating the cortex or paracortex; SNIL 3 = melanoma infiltrating the medulla or capsule. We classified 338 SN-positive patients according to the non-metric SNIL. Using Kaplan–Meier estimates and Cox models, recurrence-free survival (RFS), melanoma-specific survival (MSS) and nodal basin recurrence rates were analyzed. The median follow-up time was 75 months. The SNIL divided the SN-positive population into three groups with significantly different RFS, MSS, and nodal basin recurrence probabilities. The MSS of patients with SNIL 1 was virtually identical to that of SN-negative patients, whereas outgrowth of the metastasis from the parenchyma into the fibrous capsule or the medulla of the lymph node indicated a very poor prognosis. Thus, the SNIL may help to better assess the benefit-risk ratio of adjuvant therapies in patients with different SN metastasis patterns.

## Introduction

The histopathologic status of the sentinel lymph node (SN) is a powerful prognostic factor for patients with primary cutaneous melanomas [1]. While ~90% of the SN-negative patients survive in the long term, patients with lymph node metastasis are at increased risk of recurrence and death. In recent years, adjuvant immunotherapies and targeted therapies using anti-CTLA-4 antibodies, anti-PD-1 antibodies, or BRAF/MEK inhibition have led to improved recurrence-free survival (RFS) in melanoma patients with fully resected nodal metastases [2–4]. However, the underlying studies included only node-positive patients with metastatic deposits ≥1 mm in diameter. It has been shown that with surgery alone, some subgroups of patients with low SN tumor burden survive at a similarly high percentage as SN-negative patients [5–7]. This bears a risk that patients with very small tumor deposits are unnecessarily up-staged and thus given inaccurate prognostic information or unnecessary adjuvant therapy. One should keep in mind that modern adjuvant therapies sometimes cause significant toxicities.

An adequate indication for adjuvant therapy of melanoma therefore requires an N-staging that takes into account the tumor load in the SN. It has become increasingly clear that completion lymph node dissection (CLND) after tumor-positive SNB does not prolong melanoma-specific survival [8, 9]. As a result, the use of CLND has decreased significantly, which reduces the importance of the number of lymph node metastases as a prognostic parameter for patients with clinical occult disease. Some SN tumor burden-based prognostic models offer feasible alternatives, for example the Rotterdam classification [10], the S-classification [11], the number of mitoses within SN metastasis [12], the microanatomic metastasis location according to Dewar [13], the number of SN metastatic foci [14], the SN cross-sectional area involved by melanoma [14–16], and the presence of extranodal involvement [14, 17] (for review see [18]).

According to Willard–Mack [19], lymph nodes consist of multiple lymphoid lobules surrounded by lymph-filled sinuses and enclosed by a capsule. The follicles and interfollicular cortex of all adjacent lobules within a lymph node constitute the superficial cortex, their deep cortical units constitute the paracortex and their medullary cords and medullary sinuses constitute the medulla. We sought to determine whether a prognostically relevant classification could be established from the pattern of metastatic infiltration of these anatomically and immunologically defined substructures of the SN and developed the sentinel node invasion level (SNIL) as non-metric staging system.

## Materials and methods

### Patients

Utilizing our electronic database, we identified 1250 consecutive patients who underwent SNB for primary cutaneous melanoma between 1998 and 2017. Clinical and histological data were collected prospectively. Indications for SNB were a Breslow thickness of ≥1 mm or <1 mm if the Clark level was ≥IV or if regression, ulceration or nodal tumor growth were documented. Satellite metastases were no contraindication for SNB. We excluded 23 patients in whom a SN could not be detected during surgery.

### SN mapping technique

Radioactive lymph nodes that appeared first during lymphoscintigraphy or displayed an afferent vessel were defined as SNs. During surgery, lymph nodes that stained blue or that emitted ≥10% of the radioactive signals of the most radioactive lymph node were also defined as SNs [20].

### Pathological SN assessment

Prospectively defined protocols for pathologic workup and reporting of SNs were used as previously described [21]. Lymph nodes were cut parallel to the longest axis into slices ~1 mm thick and embedded in paraffin. Four microtome sections (3 μm thick) were made from each slice. The first was stained with hematoxylin-eosin; the subsequent for the immunohistochemistry with S100 (S100A1 and S100B expressed by melanoma cells; Dako Germany, clone: S-100, dilution 1: 3000), HMB-45 (human melanoma black/premelanosome protein 17; Dako Germany, clone: HMB45, dilution 1: 200), and Melan A (melanoma antigen recognized by T cells/melanocyte antigen; Zymed USA, clone: A 103/M2–7C10/M2–9E3, dilution 1: 200).

**Fig. 1:**
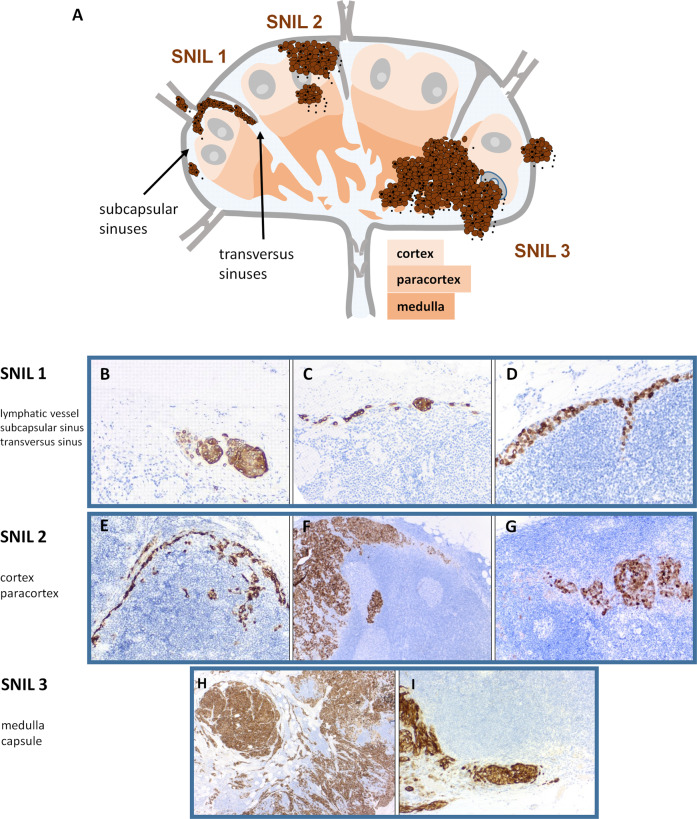
Microanatomic melanoma metastasis patterns within sentinel nodes. Upper part: (**A**) schematic figure of the sentinel node invasion level (SNIL); Pathologic figures: SNIL 1: (**B**) metastasis within a capsular lymphatic vessel, (**C**) metastasis in the subcapsular sinus, (**D**) metastasis in the subcapsular and transverse sinus without infiltration of cortex structures; SNIL 2: (**E**) metastasis in the subcapsular sinus with infiltration of cortical lymph node structures, (**F**) metastasis in the subcapsular and transverse sinus with infiltration of cortical lymph node structures metastasis within the cortex, (**G**) metastasis in the paracortex; SNIL 3: (**H**) metastasis infiltrating the cortex and medulla. **I** Metastasis infiltrating the capsule of the sentinel node. Note that isolated tumor cells within capsular lymph vessels were counted as initial metastasis and not as capsular invasion.

Melanoma cells, nevus cells and pigmented histiocytes were meticulously differentiated based on anatomic localization, cytological, and immunohistochemical criteria. According to best practice guidelines for evaluation of lymph nodes [22], our protocol required reporting of metastatic infiltration of the following structures for each SN: SN capsule, intracapsular lymph vessels, subcapsular sinus, centripetally directed transverse sinuses, cortex, paracortex, medulla, and capsule. The SNIL was formed with regard to the “deepest” tumor-affected structure in this order. Using the SN that displayed the highest tumor burden, we defined the following, functionally plausible tumor burden categories (Fig. 1):

SNIL 0 = SN-negative, no tumor cell within the SN; SNIL 1 = melanoma cells confined to intracapsular lymph vessels, subcapsular or transverse sinuses (no parenchymal metastasis); SNIL 2 = melanoma cells infiltrating the cortex or paracortex (parenchymal metastasis); SNIL 3 = metastasis breaking out from the parenchyma into medulla or capsule of the SN.

We classified tumor deposits within the subcapsular sinuses and the centripetally directed transverse sinuses as SNIL 1 only when there was a smooth border with the parenchyma and no melanoma cells infiltrated the adjacent cortex. Tumor cells exclusively in intracapsular lymphatic vessels were also counted as SNIL 1. We defined capsular invasion as partial or complete capsular destruction as a result of the expansion of intranodal melanoma metastasis.

The SN tumor burden was also classified according to the S-classification, based on the maximum distance of intranodal melanoma cells from the interior margin of the nodal capsule (tumor penetrative depth (TPD) (<0.3 mm; 0.3 mm − 1 mm; >1 mm) [11], and according to the Rotterdam criteria [23], based on the maximum diameter of the largest metastasis (MTD) (<0.1 mm; 0.1 mm − 1 mm; >1 mm). Our data collection sheet is visible as Supplementary Material. Patients with full nodal staging including complete lymph node dissection (CLND) were classified according to the *N* category of the 8th edition of the AJCC *N* classification [5].

### Statistical analyses

Statistical calculations were performed using Statistica (Version 13, TIBCO Software). Figures were created with the software R (version 3.6, www.r-project.org). We applied *t* tests or the Mann–Whitney *U* test as appropriate. Correlations of the SNIL with clinical and pathological parameters were assessed with Spearman’s correlation coefficients. Correlations were defined as weak (−0.3 < *r*_s_ < 0.3), moderate (−0.5 to −0.3 or 0.3 to 0.5) strong (−0.9 to −0.5 or 0.5 to 0.9), or very strong (−1 to −0.9 or 0.9 to 1). All survival times were calculated from the date of excision of the primary melanoma. Follow-up time, MSS, RFS, and nodal basin recurrence rates were calculated using Kaplan–Meier estimates and compared using log rank tests. Nodal basin recurrence was defined as any evidence of recurrent disease within the surgical bed of the dissection (nodal and non-nodal) including relapses, which occurred after distant metastases had become apparent. Univariate Cox models were used to assess metric risk factors. Survival times by SNIL groups were tested with multivariate Cox models; relative risks were adjusted for the classification-relevant prognostic factors Breslow thickness and ulceration. In addition, age was included in the multivariate model because it was the only significant factor in univariate analyses that was not related to SN tumor burden.

### Follow-up

The patients were monitored routinely at 3-month intervals for the first 5 years and every 6 months for the next 5 years, in accordance with the valid guidelines in Germany [24].

## Results

### Patient cohort

The most common site of the leading nodal basin was axilla (631 patients (51.4 %)), followed by groin (424 patients (34.6 %)), neck (171 patients (13.9 %)), and interval nodes (1 patient (0.1 %)). The SN positivity rate was 28%. The patient characteristics according to SN status are shown in Table 1. The median follow-up was 75 months.

**Table 1:** Baseline characteristics of patients according to the sentinel node invasion level.

Feature	Overall population with SN biopsy	SN-negative SNIL 0	SN-positive SNIL 1	SN-positive SNIL 2	SN-positive SNIL 3	*r*_s_ strength of correlation)	*P*
No. of patients	1227	884	75	198	65		
Median follow-up/months	75	72	90	92	64		
Median age/years (IQR)	61 (47–72)	61 (49–72)	56 (38–73)	58 (44–70)	59 (50−71)		ns
Female	603 (49.1%)	447 (50.6%)	33 (44%)	95 (45.8%)	26 (40%)		ns
Median breslow thickness/mm (IQR)	1.7 (1.1–3.0)	1.5 (1−2.5)	1.6 (1.1–3.1)	2.2 (1.3–3.6)	3.8 (2.9−7)	0.26 (weak)	<0.001
Mean breslow thickness/mm (*SD*)	2.4 ± 2.2	2.1 ± 2.0	2.6 ± 2.3	2.8 ± 2.1	5.0 ± 3.3		
pT1 (≤1 mm)	276 (22.7%)	227 (25.9%)	18 (24.3%)	30 (15.2%)	1 (1.54%)		
pT2 (1.01–2 mm)	390 (32.0%)	363 (41.4%)	27 (36.5%)	59 (29.8%)	8 (12.31%)		
pT3 (2.01–4 mm)	286 (23.5%)	179 (20.4%)	16 (20.3%)	67 (33.8%)	24 (36.92%)		
pT4 (>4 mm)	196 (16.1%)	108 (12.3%)	14 (17.3%)	42 (21.2%)	32 (49.23%)		
Ulceration present	335 (28.5%)	200 (22.7%)	21 (28%)	73 (37.8%)	30 (61.2%)	0.18 (weak)	<0.001
Satellite metastases	38 (3.1%)	20 (2.3%)	2 (2.7%)	10 (5.1%)	6 (9.2%)	0.09 (weak)	<0.01
Benign SN nevus	170 (15.5%)	128 (16.8%)	13 (17.3%)	26 (13.4%)	3 (4.7%)	−0.06 (weak)	<0.05
Mean TPD/mm (*SD*)	1.23 ± 1.33*	0.0	0.27 ± 0.4	1.0 ± 0.8	3.0 ± 1.6	0.71 (strong)	<0.001
Mean MTD/mm (*SD*)	1.60 ± 2.29*	0.0	0.45 ± 1.0	1.1 ± 1.2	4.5 ± 3.3	0.63 (strong)	<0.001
No. of patients with CLND	191* (57.0%)	1 (0.1%)	23 (31%)	121 (61%)	47 (72.0%)	0.27 (weak)	<0.001
No. with tumor-positive CLND**	50 (26.2%)	0 (0%)	4 (18%)	25 (20.5%)	21 (44.5%)	0.21 (weak)	<0.01
Mean No. of metastatically involved nodes**		0	1.52 ± 0.9	1.69 ± 1.44	2.7 ± 1.56	0.98 (very strong)	<0.001

*P* probability, *r*_s_ Spearman’s rank correlation coefficient (interpretation of *r*_s_: weak (−0.3 to <0.3), moderate (−0.5 to −0.3 or 0.3 to 0.5) strong (−0.9 to −0.5 or 0.5 to 0.9) or very strong (−1 to −0.9 or 0.9 to 1).), *SD* standard deviation, *SN* sentinel lymph node, *SNIL* sentinel node invasion level, *IQR* interquartile range, *TPD* tumor penetrative depth, *MTD* maximum diameter of the largest SN metastasis, *CLND* completion lymph node dissection.

*Only SN-positive patients.

**Only patients with CLND.

### Formation of prognostic groups

**Fig. 2:**
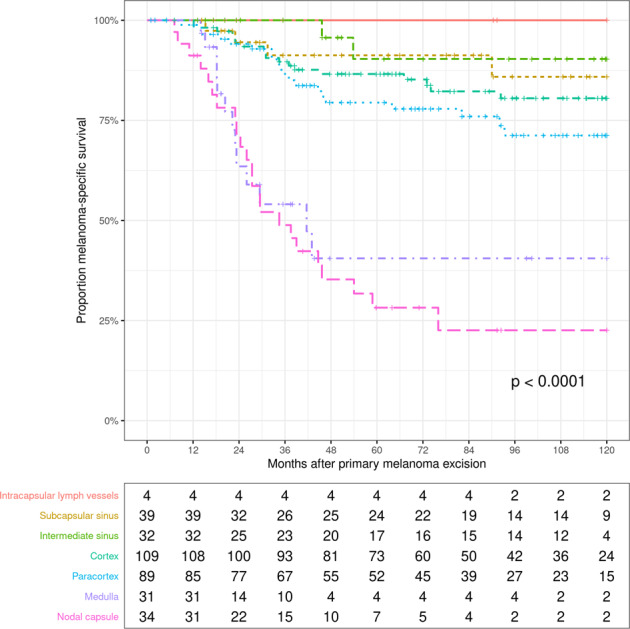
Melanoma-specific survival according to the deepest microanatomic structure infiltrated by melanoma cells. Melanoma deposits in intracapsular lymph vessels or sinuses of the SN had a favorable prognosis. Compared with melanoma infiltration of the cortex, metastasis to the paracortex indicated a somewhat decreased MSS (*P* = 0.07). Infiltration of the SN medulla or (additional) invasion of the capsule indicated very poor MSS.

The MSS rates according to the microanatomic SN substructures infiltrated with melanoma are depicted in Fig. 2. The deeper the tumor cells invaded into the SN, the worse the prognosis was. Of the 344 SN-positive patients, 338 were classified according to the SNIL. Of these, 22 % had early invasion of melanoma cells confined to intracapsular lymph vessels, subcapsular or transverse sinuses (SNIL 1), 59% had melanoma infiltration into the cortex or paracortex (SNIL 2), and 19% had melanoma infiltration including the medulla (*N* = 31), the capsule (*N* = 16), or both (*N* = 18) (SNIL 3). As shown in Table 1, the SNIL correlated significantly with the main risk factors of primary melanoma, i.e., Breslow thickness, ulceration, TPD, MTD and the number of metastatically involved lymph nodes. There was a very strong correlation of the SNIL with the S-classification (*r*_s_ = 0.99, *P* < 0.001), the Rotterdam system (*r*_s_ = 0.99, *P* < 0.001) and the AJCC N classification (*r*_s_ = 0.93, *P* < 0.001).

### Survival rates according to the SNIL

**Fig. 3:**
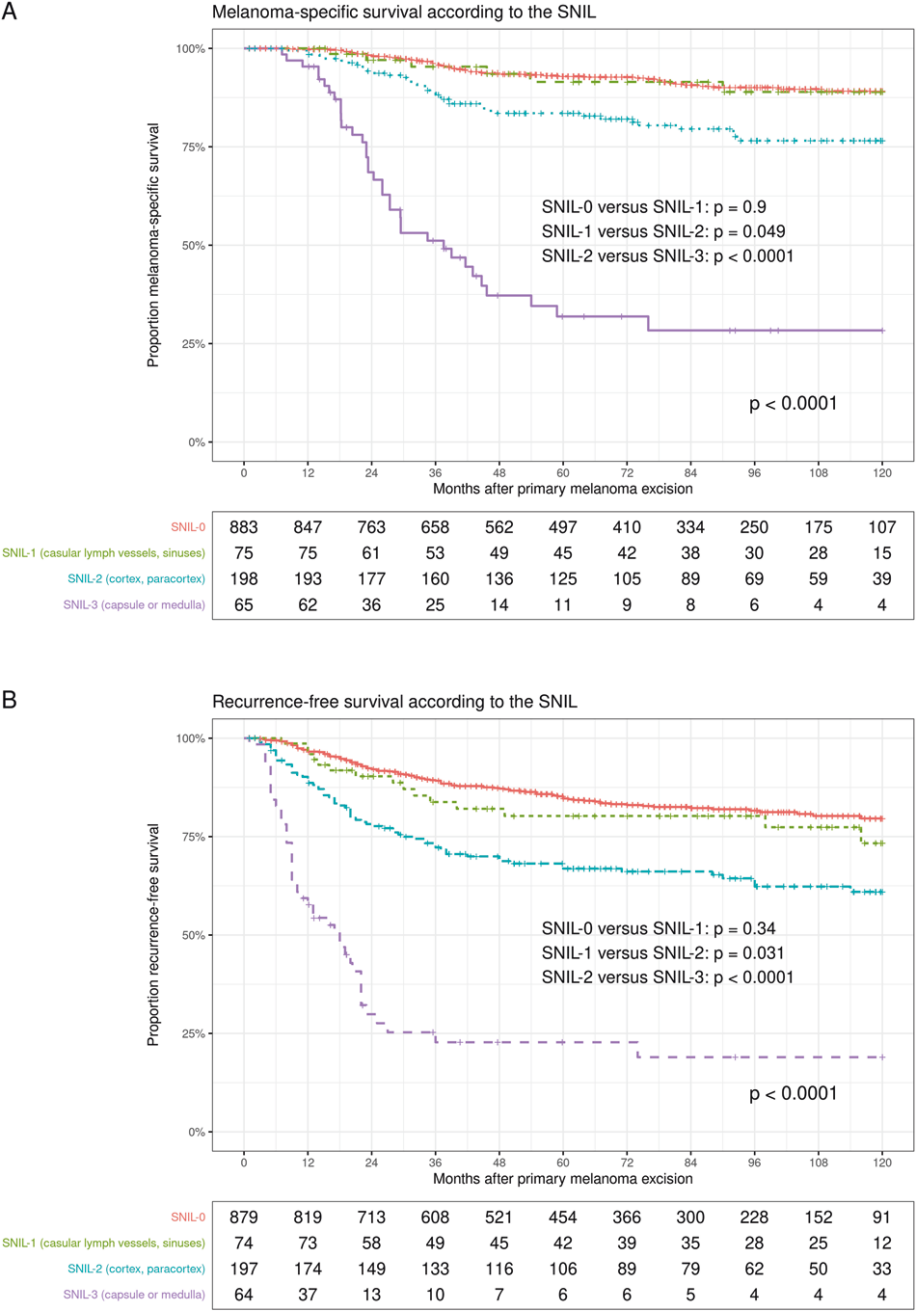
Survival rates according to the SNIL. **A** Melanoma-specific survival rate according to the sentinel node invasion level (SNIL). **B** Recurrence-free survival according to the SNIL.

By using the SNIL, we could identify three groups of SN-positive patients with significantly different prognosis on univariate analyses. The 5-year MSS rates for patients classified as SNIL 1, SNIL 2, and SNIL 3 were 91.4%, 83.5%, and 31.7%, respectively (*P* < 0.0001, Fig. 3A). On univariate analyses, the metric parameters of SN tumor-burden MTD (hazard ratio (HR) 1.19, 95% Confidence Interval (95% CI) 1.13–1.26, *P* < 0.0001) and TPD (HR 1.49, 95% CI 1.33–1.66, *P* < 0.0001) were very significant as continuous parameters. Breslow thickness (HR 1.23, 95% CI 1.18−1.29, *P* < 0.0001), ulceration (HR 3.58, 95% CI 2.59–4.94, *P* < 0.0001) and age (HR 1.02, 95% CI 1.01−1.03, *P* = 0.009) were also significant.

The same factors that determined the MSS were significant for the RFS. The RFS curves according to SNIL are shown in Fig. 3B. After adjustment for Breslow thickness, ulceration and age, the SNIL turned out to be an independent predictor of MSS and RFS in the SN-positive subpopulation (Table 2A, B).

**Table 2:** Multivariate Cox regressions analyses with focus on the SNIL (330 SN-positive patients with complete datasets).

Factor	Reference	Hazard ratio	95% Confidence interval	*P*
A. SNIL – Melanoma-specific survival				
SNIL 2	SNIL 1	2.21	0.94–5.22	0.070
SNIL 3	SNIL 1	9.10	3.69–22.19	<0.0001
Breslow	/mm	1.12	0.98–1.14	0.135
Ulceration	Absent	1.64	1.01–2.68	0.047
Age	/Year	1.01	1.00–3	0.094
B. SNIL – Recurrence-free survival				
SNIL 2	SNIL 1	1.76	1.18–4.04	0.01
SNIL 3	SNIL 1	6.92	3.53–13.57	<0.001
Breslow	/mm	1.09	1.03–17	0.01
Ulceration	Absent	1.64	1.10–2.43	0.01
Age	/Year	1.009	1.00–1.02	0.08
C. SNIL – Nodal basin recurrence-free survival				
SNIL 2	SNIL 1	2.71	1.04–7.07	0.042
SNIL 3	SNIL 1	9.90	3.56–27.64	<0.0001
Breslow	/mm	1.08	0.99–1.18	0.096
Ulceration	Absent	1.61	0.92–2.81	0.098
Age	/Year	1.03	1.01–1.05	0.005
CLND	No CLND	0.59	0.34–1.03	0.066

*CLND* complete lymph node dissection, *P* probability, *SNIL* sentinel node invasion level.

### Nodal basin recurrence rates of SN-positive patients according to the SNIL

Using univariate analyses, the SNIL strongly predicted the probability of nodal basin recurrence (*P* < 0.001). In the group classified as SNIL 1, the estimated 5-year nodal basin recurrence rates for patients with and without CLND were very low (0.0% vs. 3.1%, respectively, *P* = 0.76). Patients with SNIL 2 seemed to benefit from CLND with respect to nodal basin tumor control (recurrence rates 7.8% vs. 24.8%, respectively, *P* = 0.002). The estimated 5-year nodal basin recurrence rates for SNIL 3 patients were high with and without CLND (42.3% vs. 52.4%, respectively, *P* = 0.33). Using multivariate analysis, the significance of the SNIL for nodal basin recurrence was confirmed (Table 2C).

### Subgroup analyses according to the SNIL

#### Common analysis of the low-risk categories SNIL 0 and SNIL 1

We identified 908 patients classified as SNIL 0 or SNIL 1 with complete datasets for multivariate analyses. As shown in Table 3, the presence of SN metastases confined to intracapsular lymphatics, subcapsular sinuses, or transverse sinuses (SNIL 1) did not significantly affect MSS or RFS even after adjustment for established risk factors. Breslow thickness, ulceration, and age remained significant.

**Table 3:** Multivariate Cox analysis of melanoma-specific and recurrence-free survival (908 patients with complete datasets classified as SNIL 0 or SNIL 1).

Factor	Reference	Hazard ratio	95% Confidence interval	*P*
Melanoma-specific-survival				
SNIL 1	SN-negative	0.94	0.41–2.18	0.89
Breslow	/mm	1.17	1.07–1.28	<0.001
Ulceration	Absent	2.74	1.66–4.53	<0.001
Age	/Year	1.01	1.00–1.04	0.03
Recurrence-free survival				
SNIL 1	SN-negative	0.99	0.54–1.79	0.97
Breslow	/mm	1.19	1.12–1.26	<0.001
Ulceration	Absent	2.27	1.59–3.22	<0.001
Age	/Year	1.01	1.00–1.03	0.01

*P* probability, *SNIL* sentinel node invasion level.

#### Analysis of the low-risk category SNIL 1

On average, the SNIL 1 category included cases with deeper penetration of the metastasis into the SN than the s1 category (mean TPD 0.28 mm ± 0.4 vs. 0.12 mm ± 0.09 mm) and larger SN tumor deposits than the Rotterdam 1 category (mean MTD 0.45 ± 0.96 mm vs. 0.05 mm ± 0.03 mm).

Figure 3 demonstrates that the survival rates of the groups with SNIL 0 and SNIL 1 were very similar with respect to MSS and RFS, although the 75 patients with SNIL 1 tended to have thicker primary melanomas compared with the SN-negative patients (*P* = 0.07). On univariate analyses, Breslow thickness (HR 1.43, 95% CI 1.11–1.84, *P* = 0.006) and ulceration (HR 13.2, 95% CI 1.59–112.88, *P* = 0.004) were significant factors within the SNIL 1 group, whereas the metric parameters of SN tumor-burden MTD (*P* = 0.24) and TPD (*P* = 0.83) were not significant.

#### Analysis of the intermediate-risk category SNIL 2

With respect to MSS, Breslow thickness (HR 1.16, 95% CI 1.02–1.32, *P* = 0.024) and ulceration (HR 2,15, 95% CI 1,14−4,02, *P* = 0.017) remained significant on univariate analyses. MTD (HR 1.19, 95% CI 0.99–1.42, *P* = 0.059) tended towards significance, age (*P* = 0.170) and TPD (*P* = 0.643) were non-significant.

#### Analysis of the high-risk category SNIL 3

In univariate analyses of the SNIL 3 group, none of the melanoma-related factors examined above was significant for MSS. However, increasing age indicated a higher probability of melanoma recurrence (*P* = 0.017) (detailed results not shown).

#### AJCC N category, Rotterdam classification and S-classification

**Fig. 4:**
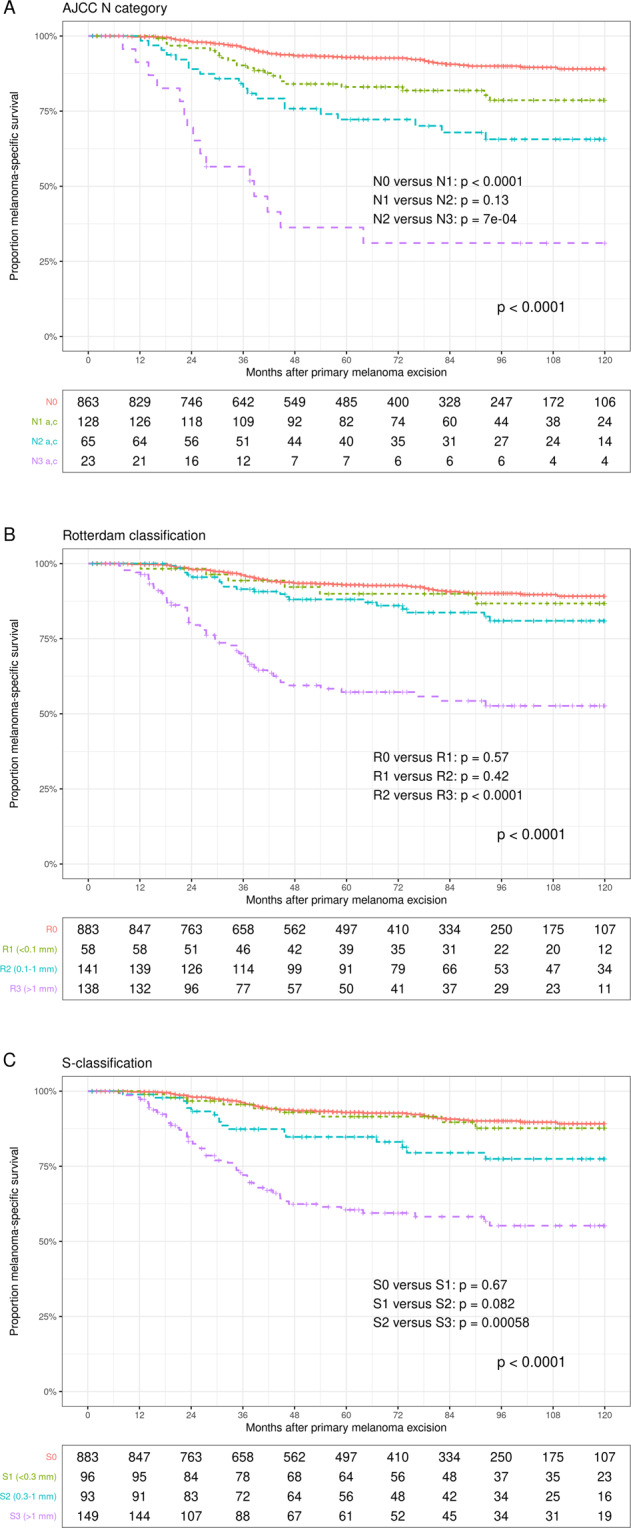
Melanoma-specific survival according to the AJCC N category, the Rotterdam system, and the S-classification. **A** Melanoma-specific survival of patients with clinically unsuspicious regional lymph nodes according to the AJCC N category (SN-positive patients without CLND excluded); **B** melanoma-specific survival according to the Rotterdam classification based on the maximum diameter of the largest tumor lesion (<0.1 mm; 0.1 mm − 1 mm; >1 mm); **C** melanoma-specific survival S-classification based on the maximum distance of intranodal melanoma cells from the interior margin of the nodal capsule (<0.3 mm; 0.3 mm − 1 mm; >1 mm).

We stratified our sample according to these classifications (Fig. 4). In contrast to the AJCC N category, the SN tumor burden-based classifications were each able to identify a low-risk group of SN-positive patients (s1, R 1). This was confirmed in multivariate analyses with analogous modelling approach as used in Table 3 (results not shown). However, the low-risk groups (R1, s1) did not differ significantly with regard to MSS from the adjacent intermediate groups (R2, s2) after adjustment for Breslow, ulceration, and age. Only the 1.0-mm cut-offs were independent predictors for the MSS in both the S-classification and the Rotterdam system (Table 4).

**Table 4:** Multivariate Cox regression analyses for melanoma-specific survival of stage III patients: comparison of AJCC N category, Rotterdam classification, and S classification.

Factor	Reference	Hazard ratio	95% Confidence interval	*P*
AJCC N category^a^ (210 complete datasets)				
N2	N1	1.55	0.856–2.81	0.146
N3	N1	3.70	1.86–7.36	0.0002
Breslow	/mm	1.11	1.02–1.20	0.014
Ulceration	Absent	1.50	0.84–2.67	0.168
Age	/Year	1.01	0.991–1.03	0.284
Rotterdam classification (329 complete datasets)				
Rotterdam 2	Rotterdam 1	1.30	0.55–3.07	0.552
Rotterdam 3	Rotterdam 1	3.34	1.50–3.07	0.003
Breslow	/mm	1.09	1.03–2.71	0.027
Ulceration	Absent	1.67	1.01–2.68	0.038
Age	/Year	1.01	0.99–1.02	0.245
S-classification (330 complete datasets)				
s 2	s 1	1.69	0.77–3.72	0.189
s 3	s 1	3.45	1.73–6.90	0.0004
Breslow	/mm	1.11	1.03–1.19	0.005
Ulceration	Absent	1.64	1.00–2.67	0.049
Age	/Year	1.01	1.00–1.02	0.168

*P* probability, *SNIL* sentinel node invasion level.

^a^Only patients with full nodal staging included.

## Discussion

The SNIL is a non-metric stratification tool that considers the metastatic infiltration of various anatomically and functionally defined substructures of SNs. The order used for the SNIL (intracapsular lymph vessels, sinuses, cortex, paracortex, medulla, and nodal capsule) reflects the natural route of metastasis spread. The Dewar classification is another non-metric staging system, which distinguish the following intranodal metastasis patterns: subcapsular, parenchymal, combined (subcapsular and parenchymal), extensive confluent, and extensive multifocal [13]. Unlike the Dewar criteria, we aimed at an exact anatomic localization of the intranodal tumor burden by describing metastatic involvement of clearly defined anatomic substructures of the SN. This required refining the term “subcapsular”. Intranodal sinuses are lined by lymphatic endothelial cells, which do not form a clear boundary but appear as a net-like structure [25]. We classified only clearly intrasinusoidal tumor deposits with a smooth border to the parenchyma as SNIL 1; any infiltrating melanoma cells within the adjacent cortex were counted as SNIL 2. Importantly, SNIL 1 also included centripetally directed melanoma deposits within the transverse sinuses (Fig. 1A, C). The question was raised as to whether these tumor cell processes, which progress to the center of the lymph node, correspond to subcapsular or parenchymal metastases [26]. We could show that metastasis extension within the transverse sinus was associated with favorable prognosis (Fig. 2). In the AJCC N category, isolated tumor cells in intracapsular lymph vessels are considered lymph node metastases [5]. Logically, we counted the rare cases of isolated tumor cells within intracapsular lymph vessels as SNIL 1. None of the four affected patients died. By contrast, infiltration of the fibrous lymph node capsule was an indicator of very poor prognosis. A study by Meier et al. demonstrated a worsened RFS with infiltration of the SN capsule [17]. We can show here for the first time that a metastatic infiltration of the SN medulla is associated with a similarly poor prognosis.

As shown by multivariate analyses, the non-metric SNIL can distinguish three groups of SN-positive patients with significantly different prognosis. The MSS of patients with SNIL 1 was virtually identical to that of SN-negative patients. An exclusively intrasinusoidal metastasis might reflect sufficient antitumoral immunity or an inability of other causes of the tumor to invade the nodal parenchyma. Breslow thickness and ulcerations remained highly significant risk factors in the low-risk category. However, we were not able to demonstrate prognostic significance of the maximum metastasis diameter for patients with SNIL 1. Borgognoni et al. [27] indirectly supported these results. They did not find a relation between the size of subcapsular metastases and tumor involvement of CLND specimens.

Also within the intermediate SNIL 2 group, Breslow and ulceration remained significant for MSS; MTD tended towards significance. The MSS of SNIL 3 patients was so poor that melanoma-associated prognostic factors became rather insignificant.

With regard to its use for surgical purposes, the SNIL was able to predict the probability of nodal basin recurrence. Only the patients classified as SNIL 2 seemed to benefit from CLND with respect to nodal basin tumor control.

According to recent guidelines, adjuvant targeted therapies or immunotherapies should be offered to patients with resected stage IIIA/B/C/D melanomas [28]. However, there are no data from controlled trials regarding the benefit in cases with minimal SN tumor burden. Single melanoma cells detected by immunohistochemistry were not associated with decreased survival [29, 30]. Moreover, several authors have assumed that SN micrometastases below a certain threshold size do not worsen prognosis. Van Akkooi et al. suggested that patients with submicrometastases with a diameter of <0.1 mm should be regarded as SN-negative. [6] Several studies supported this statement [17, 31], while others did not [32–34]. The site of such submicrometastases seems to be important. Deeper, parenchymal location worsened survival [35]. In some types of cancer, including breast cancer, nodal tumor deposits below 0.2 mm are considered N0 [36]. According to the AJCC melanoma database, the long-term survival rate of patients with SN metastases <0.2 mm was excellent (96%) [5]. On the other hand, Scheri et al. demonstrated decreased survival in melanoma patients with nodal tumor deposits <0.2 mm [32]. In agreement with van der Ploeg et al. [7], we found that a tumor penetrative depth of <0.3 mm was associated with a survival rate similar to that of SN-negative patients. Again, this contrasts with other studies reporting lower survival rates [17, 34, 37].

The differences in pathological protocols may have contributed to these inconsistent results [26]. It has been shown that the detection of small tumor deposits can indicate larger tumor nests outside the sectional plane [34, 38]. The definition a low-risk N category therefore requires standardized and sufficiently comprehensive pathology protocols. At our facility, a comprehensive pathology study was performed [21], which exceeded the usual standards.

As with SNIL 1, the MSS of the low-risk Rotterdam and S classification groups (R1, s1) was also very similar to that of SN-negative patients. However, similar to Meier’s observation [17], no significant differentiation from the adjacent intermediate-risk groups (R2, s2) was achieved by multivariate analyses (Table 4). We could only confirm the significance of the 1-mm cutoffs in the Rotterdam classification and the S-classification [12, 39].

The relatively long follow-up period (median 90 months for the SNIL 1 group) is a strength of the present study. It is known that a minimal tumor burden requires longer follow-up times. In contrast to many previous studies dealing with this topic, we performed multivariate analyses and delivered MSS rates, including for our own SN negative group. Our work also has some limitations. Although microanatomical SN metastasis patterns, primary tumor parameters and survival outcomes were collected prospectively, SNIL grouping was done, retrospectively. Ideally, the SNIL, like any SN tumor burden classification, should be validated in a controlled multicenter study, which should also address reproducibility between observers and the influence of different protocols of pathological work-up. It could be argued that the overall survival rates reported in our study might be biased by immune checkpoint therapies or targeted therapies that have been routinely administered for non-resectable recurrences since 2011. However, the SNIL was able to distinguish prognostic groups in terms of RFS and nodal basin recurrence rates. These results are clearly not biased as patient enrollment in the present study ended prior to approval of anti-PD-1 antibodies or BRAF/MEK inhibition in the adjuvant indication.

## Conclusions

The main contribution of our study is that the SNIL can delineate three independent risk groups of SN-positive patients in terms of MSS, RFS, and nodal basin tumor control without the need for distance measurements under the microscope. We sought to define more precisely the incipient lymph node metastasis of melanoma. The SNIL 1 category included not only metastases restricted to the subcapsular sinus, but also melanoma cells within the intracapsular lymphatic vessels and within the centripetally directed transverse sinus. These patterns of early metastasis were not associated with worse melanoma-specific survival compared with SN-negative patients. The metric SN tumor burden parameters TPD and MTD were not significant for patients classified as SNIL 1. According to our data, adjuvant therapy is not warranted in SNIL 1. However, since Breslow and ulceration remained significant, they must be considered. In contrast to the metric SN classifications, which use metastatic extension of ≥1 mm to delineate the worst prognostic group, SNIL 3 was able to delineate a group with even worse prognosis, in which tumor infiltration of the medulla or capsule of the SN is present. Thus, SNIL can contribute to the development of future tumor burden-based N staging in melanoma. The SNIL may be also helpful to more accurately assess the benefit-risk ratio of adjuvant therapies such as BRAF/MEK inhibition or checkpoint blockade.

## Supplementary information

Data collection sheet

## Data Availability

The datasets used and analyzed during the current study are available from the corresponding author on reasonable request.
