# Supplementary material for: The sentinel node invasion level (SNIL) as a prognostic parameter in melanoma
Source: Mod Pathol. 2021 Jun 15;34(10):1839–49. doi: 10.1038/s41379-021-00835-5 (PMC8443441; doi:10.1038/s41379-021-00835-5)
Supplement: Supplementary file 1 — Data collection sheet [file 41379_2021_835_MOESM1_ESM.docx]

**Pathologic evaluation of sentinel lymph nodes of malignant melanoma**

**Data collection sheet**

**Miniabstract**

The sentinel node invasion level (SNIL) was defined as follows: SNIL 1 = melanoma cells confined to intracapsular lymph vessels, subcapsular or transverse sinuses; SNIL 2 = melanoma infiltrating the cortex or paracortex; SNIL 3 = melanoma infiltrating the medulla or capsule. The SNIL divided the SN-positive population into three groups with significantly different prognosis.

Data collection sheet (Please fill out a form for each SN)

| **Patient data:** | | | |
| --- | --- | --- | --- |
| **Internal case number:** | | | |
| 1. SN No.: | No. radioactive counts: | | blue colored: yes no |
|  | | | |
| **Size of lymph node (length x width x height) in mm:** | |  | |
|  | |  | |
| **H&E stainings:** | | negative positive | |
| **Immunohistochemistry** | | negative positive | |
| S-100 | | negative positive | |
| HMB-45 | | negative positive | |
| Melan A | | negative positive | |
| Other Antibody: | | negative positive | |
|  | |  | |
| SN melanocytic nevus: | | yes no | |
| **SN status** | | **negative positive** | |
| Number of metastatic foci | | 1 2-5 disseminated | |
| Single cells only | | yes no | |
|  | | | |
| **Deepest SN structure invaded with melanoma cells** | | intracapsular lymph vessels : yes no  subcapsular sinus: yes no  transverse sinuses: yes no  cortex: yes no  paracortex: yes no  Medulla: yes no  Capsule: yes no | |
| Maximum diameter of the largest metastasis | | mm | |
| Tumor penetrative depth from the inner margin of the SN capsule: | | mm | |

SN status (Summary)

| **Patient data:** | | |
| --- | --- | --- |
| **Internal case number:** | | |
| **(B)** Classification of nodal status: **SN**-**negative SN-positive** | | |
| No. of SNs excised: | | |
| No. of tumor-positive SNs: |  |  |
| ↓ **Please consider only the SN with the highest tumor burden:** ↓ | | |
| SNL invasion level (SNIL): | Maximum diameter of the largest metastasis:  mm | Invasion depth from the inner margin of the capsule:  mm |
| SNIL1:  (Intracapsular lymph vessels  subcapsular sinuses  transverse sinuses)  SNIL2:  (Cortex, paracortex)  SNIL3:  (Medullar and or capsular invasion) | Rotterdam 1 (<0,1mm)  Rotterdam 2 (0,1-1,0 mm)  Rotterdam 3 (> 1 mm) | S1: (< 0,3 mm)  S2: (0,3 mm - 1mm)  S3: (>1mm) |
